# Fabrication and Characterization of a Novel 3D-Printable Bio-Composite from Polylactic Acid (PLA) and Ruminant-Digested Corn Stover

**DOI:** 10.3390/polym17152077

**Published:** 2025-07-29

**Authors:** Siyang Wu, Lixing Ren, Jiyan Xu, Jiale Zhao, Xiaoli Hu, Mingzhuo Guo

**Affiliations:** 1 College of Engineering and Technology, Jilin Agricultural University, Changchun 130118, China; wsy@jlau.edu.cn (S.W.); 20240284@mails.jlau.edu.cn (L.R.); 2304100312@mails.jlau.edu.cn (J.X.); 2 College of Biological and Agricultural Engineering, Jilin University, Changchun 130022, China; zhaojl@jlu.edu.cn

**Keywords:** ruminant-digested fiber, PLA composites, 3D printing, circular bioeconomy, sustainable materials, fused deposition modeling

## Abstract

To address the growing demand for sustainable materials in advanced manufacturing, the objective of this study was to develop and characterize a novel 3D-printable biocomposite using ruminant-digested corn stover (DCS) as a reinforcement for polylactic acid (PLA). The methodology involved systematically optimizing DCS particle size (80–140 mesh) and loading concentration (5–20 wt.%), followed by fabricating composite filaments via melt extrusion and 3D printing test specimens. The resulting materials were comprehensively characterized for their morphological, physical, and mechanical properties. The optimal formulation, achieved with 120-mesh particles at 15 wt.% loading, exhibited a 15.6% increase in tensile strength to 64.17 MPa and a 21.1% enhancement in flexural modulus to 4.19 GPa compared to neat PLA. In addition to the mechanical improvements, the biocomposite offers an advantageous density reduction, enabling the fabrication of lightweight structures for resource-efficient applications. Comprehensive characterization revealed effective interfacial integration and uniform fiber dispersion, validating biological preprocessing as a viable method for unlocking the reinforcement potential of this abundant biomass. While the composite exhibits characteristic trade-offs, such as reduced impact strength, the overall performance profile makes it a promising candidate for structural applications in sustainable manufacturing. This research establishes a viable pathway for agricultural waste valorization, demonstrating that biological preprocessing can convert agricultural residues into value-added engineering materials for the circular bioeconomy.

## 1. Introduction

Natural fibers are increasingly recognized as promising renewable reinforcements for polymer composites, valued for their low cost, biodegradability, and favorable mechanical properties. This concept builds on a long history of using digested biomass, such as straw mixed with clay to create durable adobe bricks for construction, demonstrating the principle of valorizing agricultural residues. Heightening environmental concerns and stringent regulations have catalyzed research into sustainable alternatives to conventional synthetic materials for a wide range of industrial applications. Compared to their synthetic counterparts, such as glass or carbon fibers, these lignocellulosic materials offer distinct advantages, including low density, high specific strength, and alignment with the principles of a circular bioeconomy. Consequently, the market for natural-fiber-reinforced polymer composites (NFRPCs) is undergoing significant expansion, reflecting increasing adoption across key industrial sectors such as the automotive and construction industries [1,2,3]. Indeed, Hiremath et al. [4] note that this expansion is driven by a dual demand for performance and sustainability. Among these sources, agricultural residues represent a particularly abundant and underutilized feedstock that can effectively enhance the performance of biopolymers while potentially reducing overall material costs. This valorization strategy not only mitigates waste disposal issues but also fosters economic development in rural communities.

While conventional lignocellulosic fibers such as hemp, jute, and flax have been extensively studied, ruminant-digested corn stover (DCS) represents a novel and largely unexplored reinforcement material. This material is unique in that it undergoes a natural biological pretreatment within the ruminant digestive system, which modifies its surface chemistry and morphology in ways distinctly different from raw agricultural residues. This in situ enzymatic hydrolysis by the rumen microbiome selectively degrades amorphous hemicellulose and modifies the lignin structure, thereby preserving the mechanically robust crystalline cellulose core of the original fibers. Weimer et al. [5] hypothesized that this process could enhance fiber–matrix compatibility without the need for aggressive chemical treatments. This biological pathway stands in stark contrast to conventional thermochemical pretreatments, which, as documented by Harrison et al. [6], often require harsh reagents, consume significant energy, and generate hazardous effluent. Paradoxically, while beneficial for material science, these recalcitrant fibers are problematic in other contexts; they are known to impede anaerobic digestion, reducing methane yields in biogas facilities, and complicate manure management systems [7]. Therefore, the valorization of DCS into high-performance composites presents a compelling opportunity to transform an environmental and operational liability into a value-added product.

Fused Deposition Modeling (FDM), a dominant additive manufacturing technology, has revolutionized production by enabling the rapid fabrication of complex and customized geometries. Within the FDM landscape, polylactic acid (PLA) is the preeminent bio-based polymer, favored for its excellent printability, biodegradability, and mechanical properties. However, the widespread application of neat PLA is constrained by its inherent brittleness, poor impact toughness, and relatively high cost compared to petroleum-based commodity plastics. The incorporation of natural fibers has emerged as a viable strategy to mitigate these deficiencies. For instance, studies by Boey et al. [8] and Miedzianowska et al. [9] have demonstrated that lignocellulosic fillers can simultaneously enhance stiffness and reduce material costs. However, the successful fabrication of these composites via FDM is not trivial and depends critically on optimizing filler characteristics and processing parameters. Fiber particle size, for example, directly governs dispersion quality and interfacial adhesion. Indeed, findings from Hu et al. [10] indicate that while finer particles generally improve flow and surface finish, they may offer diminished reinforcement compared to particles with a higher aspect ratio. Similarly, fiber loading must be precisely controlled. While loadings between 10 and 20 wt.% are often cited as optimal, exceeding this threshold frequently leads to a sharp increase in melt viscosity. This can cause processing defects such as nozzle clogging, poor interlayer adhesion, and void formation, ultimately degrading the mechanical integrity of the printed part—a phenomenon documented by Leong et al. [11] and Azam et al. [12].

Building upon this context, this study investigates the development and characterization of a novel, 3D-printable biocomposite derived from polylactic acid (PLA) and ruminant-digested corn stover (DCS) particles. The novelty of this work lies in valorizing this unique, biologically pretreated agricultural residue for an advanced manufacturing application. The significance is rooted in its alignment with circular bioeconomy principles, transforming an environmental and operational liability into a sustainable, performance-enhanced material. The primary contribution of this research is the establishment of a clear processing–structure–property relationship for this new biocomposite system, providing a foundational framework for its application. To achieve this, the specific objectives are to (1) determine the optimal DCS particle size (80–140 mesh range) for achieving effective interfacial compatibility with the PLA matrix; (2) identify the ideal fiber loading concentration (5–20 wt.%) that balances mechanical reinforcement with FDM processability; (3) evaluate the physical properties of the resulting composites, including density, porosity, and water absorption; (4) comprehensively characterize the mechanical performance via tensile, flexural, and impact testing.

## 2. Materials and Methods

### 2.1. Materials

Polylactic acid (PLA) pellets (Ingeo Biopolymer 4032D) were procured from Hebei Chengtong Technology Co., Ltd. (Xingtai, China). According to the manufacturer’s specifications, this extrusion-grade PLA has a density of 1.24 g/cm^3^, a glass transition temperature of approximately 58 °C, a melting temperature range of 160–170 °C, and a melt flow index (MFI) of 7 g/10 min (210 °C, 2.16 kg). This particular grade is frequently selected for filament fabrication in Fused Deposition Modeling (FDM) due to its well-documented thermal stability and favorable rheological properties, which ensure consistent printability and interlayer adhesion [13].

The lignocellulosic reinforcement was derived from corn (cultivar: Hengyu No. 1), harvested during the autumn season from commercial farms in the Changchun agricultural region, China. Post harvest, the stover was field-dried to a moisture content of approximately 15% prior to collection. To prepare it for ingestion, the dried stover was mechanically processed through a forage chopper to achieve a uniform particle length of 2–3 cm. The biological pretreatment was performed in vivo using Simmental cattle (*n* = 12; average body weight: 650 ± 50 kg), which were housed at a commercial breeding facility under standard management protocols. The cattle were fed a total mixed ration (TMR) formulated to contain 60% corn stover on a dry matter basis, a composition consistent with regional agricultural practices for optimizing both animal health and fiber digestion. The remaining 40% of the TMR consisted of a concentrate mixture of corn grain, soybean meal, and mineral supplements. To preserve the integrity of the digested corn stover (DCS) fibers, fresh manure was collected within two hours of defecation, thereby minimizing post-excretion secondary fermentation and microbial degradation. This rapid collection protocol is considered critical, as prolonged environmental exposure can lead to aerobic decomposition that alters the fiber’s surface chemistry and compromises its reinforcing potential [14].

### 2.2. Preparation of Composite Materials

#### 2.2.1. Extraction and Sieving of Ruminant-Digested Corn Stover

The initial extraction involved washing the manure under running tap water over a stainless-steel mesh strainer (5 mm aperture) to separate the fibrous DCS components from soluble organic matter and fine particulates. This rinsing was continued until the effluent ran clear, a process that typically required 15–20 min. Subsequently, the washed fibers underwent a mild alkaline treatment to remove residual amorphous non-cellulosic constituents. The fibers were immersed in a 5% (*w*/*v*) sodium hydroxide (NaOH) solution at a solid-to-liquid ratio of 1:20 for 2 h at ambient temperature (≈25 °C) with intermittent stirring. Following this treatment, the fibers were rinsed with distilled water until a neutral pH (≈7.0) was confirmed in the effluent using pH indicator strips. This purification step was designed to remove non-cellulosic components such as proteins and lipids, thereby increasing surface roughness and exposing cellulose-rich hydroxyl groups critical for subsequent functionalization. This multi-stage preparation, involving alkaline treatment, prolonged oven drying, and subsequent high-temperature melt extrusion, also serves to effectively sterilize the fibers, mitigating any potential health concerns associated with their manure-based origin.

To address the inherent chemical incompatibility between the hydrophilic DCS fibers and the hydrophobic PLA matrix, a critical surface functionalization was performed to promote interfacial adhesion. This approach builds upon the foundational work of Gao et al. [15] and Hasan et al. [16], who demonstrated that silanization is highly effective for creating covalent linkages across the fiber–matrix interface. Accordingly, the purified fibers were treated by immersion in a 5% (*w*/*v*) solution of γ-aminopropyltriethoxysilane (KH550) prepared in an aqueous ethanol solvent (95% *v*/*v*). The pH of the solution was adjusted to ≈4.5 with acetic acid to catalyze the hydrolysis of the silane’s ethoxy groups into reactive silanols. The slurry was maintained at 50 °C for 2 h with continuous stirring to ensure a uniform deposition of the coupling agent.

Following surface modification, the DCS fibers were dried in a convection oven at 60 °C for 24 h, or until a constant mass was achieved, to eliminate residual moisture and solvent. The desiccated fibers were subsequently pulverized using a laboratory mill and fractionated by size via a standard sieve shaker equipped with 80-mesh (180 μm), 100-mesh (150 μm), 120-mesh (125 μm), and 140-mesh (106 μm) sieves. This size classification was conducted for 10 min at a medium amplitude to ensure effective particle segregation. Each resulting size fraction was collected and stored in a sealed, desiccated container at ambient temperature pending composite fabrication.

#### 2.2.2. Fabrication of PLA/DCS Composite Filaments

Composite filaments were prepared via single-screw melt extrusion to serve as the feedstock for subsequent FDM. To systematically determine the optimal particle size, it was necessary to first establish a constant fiber loading for this preliminary comparative study. A concentration of 10 wt.% was strategically chosen for this screening phase, as this loading, established by Rangappa et al. [17], is sufficiently high to assess interfacial phenomena in lignocellulosic-PLA systems, yet low enough to avoid significant rheological complications that could otherwise obscure the effects of particle geometry. Prior to extrusion, the dried PLA pellets and each respective DCS size fraction (80, 100, 120, and 140 mesh) were dry-blended with a maleic anhydride-grafted polylactic acid (PLA-g-MA) compatibilizer, which was added at a concentration of 3% by weight relative to the DCS particles. The inclusion of PLA-g-MA builds upon the work of Gonzalez-Lopez et al. [18], who demonstrated that such compatibilizers can effectively bridge the interface by forming bonds with the fiber’s hydroxyl groups while its polymer backbone entangles with the PLA matrix.

The pre-compounded mixture was introduced into the extruder’s feed hopper. The blend was conveyed by a rotating screw through a barrel with a flat temperature profile of 170 °C, a condition selected to ensure complete melting of the PLA while minimizing the potential for thermal degradation of the DCS fibers. The combination of thermal energy and mechanical shear facilitated the homogenization of the fibers within the polymer melt. The molten composite was then extruded through a circular die with a 1.75 mm diameter. Upon exiting the die, the extruded strand was immediately quenched by forced-air cooling and drawn by a calibrated puller at a constant velocity to achieve a consistent diameter of 1.75 ± 0.05 mm before being spooled for subsequent use. This fabrication protocol was repeated for each of the four DCS particle size fractions to produce distinct filament batches for comparative analysis.

#### 2.2.3. Fabrication of Test Specimens via FDM

Standardized test specimens were fabricated from the neat PLA and PLA/DCS composite filaments using FDM. Prior to fabrication, 3D models of the test specimens (conforming to ASTM D638, D790, and D256) were processed using Ultimaker Cura software (version 5.9.0) to generate the G-code toolpaths. A highly conservative set of printing parameters was used for all formulations to maximize print reliability and ensure the comparability of results. To accommodate the fibrous nature of the DCS-filled composite and eliminate extrusion issues, a 1.0 mm diameter hardened steel nozzle was employed. This larger nozzle diameter significantly reduced the clogging risk caused by DCS fibers while maintaining adequate dimensional control for the mechanical testing specimens.

Key printing parameters included a nozzle temperature of 170 °C, a build plate temperature of 65 °C, and a layer height of 0.4 mm. An extremely conservative printing speed of 8 mm/s was employed to ensure optimal material flow and maximum time for proper layer fusion. This ultra-reduced velocity, building upon strategies previously recommended by Ahmad et al. [19] and Roopavath et al. [20] for highly filled polymer systems, was critical for ensuring the extruded material had sufficient time to properly fuse with the preceding layer before solidification, thereby minimizing the formation of process-induced voids. These conservative parameters, particularly the larger nozzle diameter and slow print speed, were intentionally chosen to mitigate the risk of nozzle clogging by the fibrous composite and to ensure optimal interlayer fusion, thereby prioritizing the fabrication of structurally sound specimens for mechanical testing.

**Figure 1:**
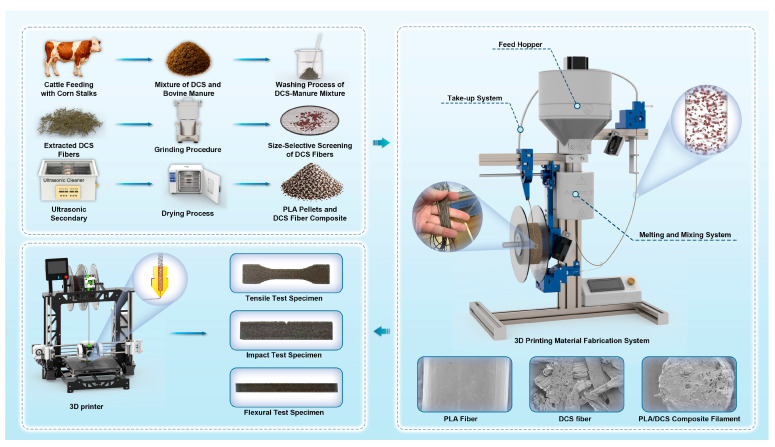
Schematic illustration of the fabrication process for PLA/DCS composite test specimens, including fiber extraction, filament extrusion, and FDM.

To produce solid parts for mechanical testing, all specimens were printed with 100% infill using a rectilinear pattern (+/−45° raster angles). Print cooling was disabled for the first 5 layers and then set to 50%. Post fabrication, specimens were stored in a desiccator pending characterization. The complete fabrication workflow is schematically summarized in Figure 1.

### 2.3. Characterization

#### 2.3.1. Mechanical Property Testing

The mechanical performance of the neat PLA and PLA/DCS composite specimens was rigorously quantified via tensile, flexural, and impact testing to establish critical structure–property relationships. Prior to characterization, all 3D-printed specimens were conditioned for a minimum of 40 h at 23 ± 2 °C and 50 ± 10% relative humidity, in strict accordance with Procedure A of ASTM D618. This conditioning protocol is particularly critical, as Le et al. [21] previously established that ambient moisture can significantly plasticize the PLA matrix and swell hydrophilic fibers, thereby altering the mechanical response. For all mechanical tests, a minimum of five replicate specimens were tested for each formulation to ensure statistical reliability.

Quasi-static tensile properties—namely, tensile strength, Young’s modulus, and elongation at break—were determined following the ASTM D638-22 standard using a universal testing machine (ZME-1010A, Lixian, Dongguan, China). Type I dumbbell-shaped specimens were tested to fracture at a constant crosshead speed of 5 mm/min. The application of these conventional testing standards to FDM parts aligns with the framework proposed by Vinal et al. [22], who validated their suitability for assessing the anisotropic properties inherent to layer-by-layer fabrication.

Flexural properties were assessed using a three-point bending configuration as specified by ASTM D790-17. This method is particularly salient for FDM components, as Rabinowitz et al. [23] established that flexural properties are exceptionally sensitive to the quality of interlayer adhesion, a critical factor in determining part integrity. Rectangular bar specimens (127 × 12.7 × 3.2 mm) were tested with a support span-to-depth ratio of 16:1 and a crosshead speed of 1.4 mm/min, which corresponds to the standard’s prescribed strain rate of 0.01 mm/mm/min for Procedure A.

To evaluate the material’s toughness, the Notched Izod impact strength was measured in accordance with Test Method A of the ASTM D256-10 standard. The selection of the Notched Izod test is consistent with the approach of Karupaiah et al. [24], who demonstrated its high sensitivity for probing interfacial adhesion quality and matrix toughness in similar lignocellulosic-biopolymer composite systems. The tests were conducted on notched rectangular specimens using a pendulum impact tester (LR-A004-5D, Langshuo, Changsha, China) equipped with a 2.7 J pendulum. The impact strength was calculated by normalizing the absorbed fracture energy by the original cross-sectional area at the notch, and the results are reported in units of kilojoules per square meter (kJ/m^2^).

#### 2.3.2. Density Measurement

The experimental density (*ρ_s_*) of the neat PLA and PLA/DCS composite specimens was precisely measured following Test Method A of ASTM D792-20, which is based on the Archimedes’ principle of liquid displacement. Measurements were performed using an analytical balance (±0.1 mg precision) equipped with a density determination kit, with five replicate specimens tested for each formulation. The characterization was conducted in a controlled laboratory atmosphere of 23 ± 2 °C, with distilled water serving as the immersion liquid.

The procedure involved first recording the mass of each specimen in air, followed by its apparent mass when fully submerged. Adhering air bubbles were meticulously removed from the specimen surface prior to the submerged measurement to prevent buoyancy-related artifacts. To minimize measurement errors arising from water absorption, the apparent mass of the immersed specimen was recorded rapidly, as stipulated by the standard. The experimental density (*ρ_s_*) was subsequently calculated for each specimen using Equation (1):

(1) $$\rho_{s}=\frac{M_{air}}{M_{air}-M_{immersed}}\times \rho_{0}$$

where *M_air_* is the mass of the specimen in air, *M_immersed_* is the apparent mass of the immersed specimen, and *ρ*_0_ is the density of the distilled water at the recorded measurement temperature.

#### 2.3.3. Porosity Features

Porosity, often referred to as void content, is a critical parameter for evaluating the internal structural integrity of 3D-printed composites, as it directly influences mechanical performance and long-term durability. The formation of such voids can occur during both filament extrusion and the subsequent FDM process, originating from factors such as entrapped air, inadequate interfacial adhesion between the fiber and matrix, and incomplete fusion between successive printed layers. As Kolken et al. [25] extensively documented, these voids function as stress concentration sites, which can prematurely initiate failure mechanisms and thereby compromise the overall strength and structural integrity of the component.

The overall porosity of the fabricated specimens was quantified by comparing the experimentally measured density with the theoretical density. The theoretical density (*ρ_t_*) of the PLA/DCS composites, which represents an idealized, void-free material, was calculated using the rule of mixtures, a standard approach for such systems as established by Pang et al. [26] and Li et al. [27]:

(2) $$\frac{1}{\rho_{t}}=\frac{W_{f}}{\rho_{f}}+\frac{W_{m}}{\rho_{m}}$$

where *W* and *ρ* represent the weight fraction and density, respectively, with the subscripts *f* and *m* denoting the DCS fiber and the PLA matrix, respectively. The density of the PLA matrix (*ρ_m_*) was taken as 1.24 g/cm^3^ per the manufacturer’s specifications, while the true density of the DCS fiber powder (*ρ_f_*) was precisely determined using a gas pycnometer. The porosity percentage (*P*) was subsequently calculated using the experimental density (*ρ_s_*), determined as detailed in Section 2.3.2, according to the following relationship:

(3) $$P=\left[\frac{\left(\rho_{t}-\rho_{s}\right)}{\rho_{t}}\right]\times 100\%$$

where *ρ_t_* is the theoretical density and *ρ_s_* is the experimental density of the composite specimen. This analytical approach provides a quantitative measure of the internal void content, offering critical insights into the quality of fiber dispersion, the degree of interfacial compatibility, and the overall efficacy of the FDM process in producing dense, well-consolidated parts.

#### 2.3.4. Water Absorption Test

The water absorption kinetics of the PLA/DCS composites were quantified to evaluate their long-term dimensional stability and susceptibility to moisture-induced degradation. This characterization is particularly critical given the inherent hydrophilicity of the lignocellulosic DCS fibers, which, as noted by Saha et al. [28], creates a fundamental incompatibility with the hydrophobic PLA matrix that directly influences the composite’s long-term durability. The water absorption rate was determined over a 168 h (7-day) period, adhering to the methodology prescribed by ASTM D570.

Prior to immersion, five replicate specimens from each composite formulation were conditioned in a convection oven at 50 ± 3 °C for 24 h to establish a consistent, anhydrous baseline state. Following this conditioning, the specimens were cooled to ambient temperature in a desiccator, after which their initial dry mass (*M_dry_*) was immediately recorded using an analytical balance (±0.1 mg precision). The specimens were then fully submerged in a thermostatically controlled bath of distilled water maintained at 23 ± 1 °C. To map the absorption kinetics, specimens were periodically removed from the bath at specified intervals (2, 4, 8, 12, 24, 48, 96, 120, and 168 h) and rapidly blotted with a lint-free cloth to eliminate surface moisture, and their wet mass (*M_t_*) was recorded before being promptly re-immersed. The percentage of water absorption (*WA*) at a given time *t* was calculated using Equation (4):

(4) $$WA=\left[\frac{\left(M_{t}-M_{dry}\right)}{M_{dry}}\right]\times 100\%$$

where *M_t_* is the mass of the specimen at immersion time *t* and *M_dry_* is the initial anhydrous mass of the specimen. This systematic procedure yielded kinetic curves for each formulation, enabling a comprehensive analysis of both the initial absorption rate and the water absorption percentage at saturation.

#### 2.3.5. Interfacial Morphology Analysis

The morphological characteristics of the raw DCS fibers and the interfacial compatibility within the PLA/DCS composite filaments were investigated via scanning electron microscopy (SEM). The analysis was conducted using a Field Emission Scanning Electron Microscope (FEI450, Thermo Fisher Scientific, Hillsboro, OR, USA) operating at an accelerating voltage of 10 kV. For a comprehensive microstructural assessment, specimens were prepared to visualize both the raw fiber material and the resulting composite structures. Sieved DCS powder was mounted directly onto aluminum stubs with conductive carbon tape to characterize its intrinsic surface features. To examine internal fiber dispersion and the quality of the fiber–matrix interface, composite filament segments were cryogenically fractured. This technique, which involves rapid fracture following immersion in liquid nitrogen, produces clean fracture surfaces devoid of the plastic deformation artifacts that typically occur at ambient temperatures. In parallel, intact filament segments were mounted to observe their external surface topography. Prior to imaging, all specimens were sputter-coated with a thin layer of gold to ensure adequate surface conductivity and mitigate charging artifacts. This multifaceted morphological investigation was crucial for comparing the interfacial adhesion associated with different DCS particle sizes, identifying the presence of voids or gaps at the interface, and ultimately selecting the optimal fiber size for subsequent detailed characterization.

#### 2.3.6. Statistical Analysis

To rigorously evaluate the significance of the observed differences among the experimental groups, a formal statistical analysis was performed. The key parameters of this analysis are summarized in Table 1. In the figures presenting these results, group means that do not share a common letter (e.g., a, b, c) are considered statistically different from one another.

## 3. Results

### 3.1. Morphological and Microstructural Analysis

#### 3.1.1. Surface Morphology of DCS Fibers

**Figure 2:**
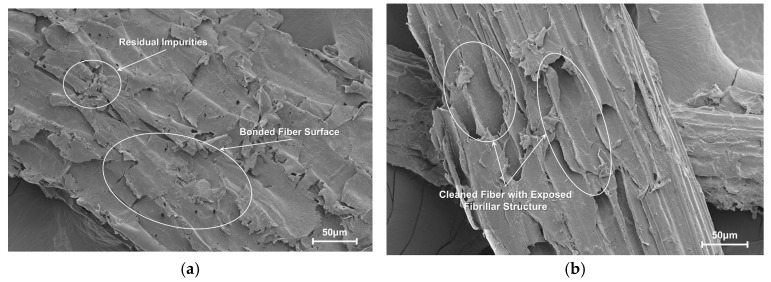
Surface morphology of DCS fibers before and after alkali treatment: (**a**) untreated fiber; (**b**) treated fiber.

Scanning electron microscopy (SEM) was employed to characterize the topographical changes in the ruminant-digested corn stover (DCS) fibers following purification. The results in Figure 2 show a significant transformation of the fiber surface. Figure 2a shows the DCS fibers after in vivo digestion and water washing, but before chemical treatment. The fiber surface is partially covered by residual impurities, likely microbial residues and organic contaminants that fill the natural grooves and create a relatively smooth surface. This smooth surface is suboptimal for composite applications as it provides limited mechanical engagement sites with the PLA matrix.

Following the 5% NaOH treatment (Figure 2b), the alkali purification successfully removes these surface impurities, revealing the fiber’s intrinsic architecture. The treatment exposes a rugged topography with distinct longitudinal grooves and defined microfibrils. This process, similar to established alkali treatments described by Poletanovic et al. [29], effectively removes non-cellulosic components and increases surface roughness. This surface modification serves two functions: enhancing mechanical interlocking with the PLA matrix through increased surface roughness, and exposing cellulose-rich hydroxyl groups necessary for subsequent silane coupling agent (KH550) grafting. The transformation from a smooth, occluded surface to a clean, textured topography is essential for achieving strong interfacial adhesion in the composite [30,31].

#### 3.1.2. Optimization of DCS Particle Size: Interfacial Morphology of PLA/DCS Composites

**Figure 3:**
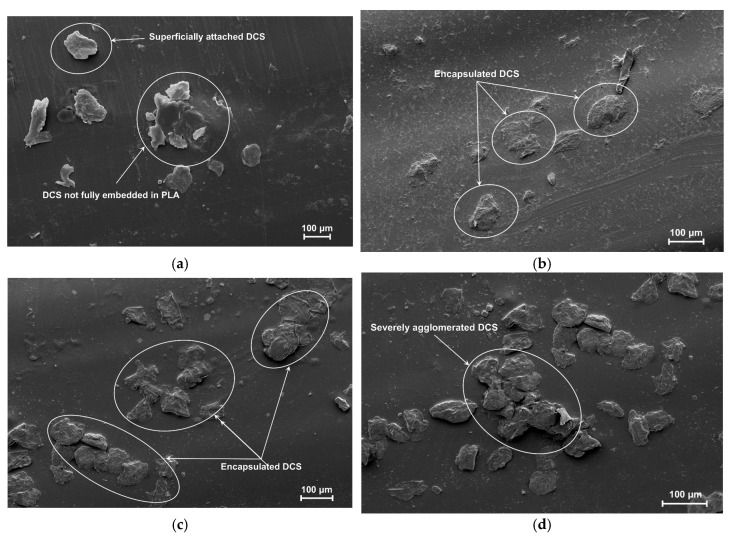
SEM micrographs of the surface of PLA/DCS composites: (**a**) 80-, **(b**) 100-, (**c**) 120-, and (**d**) 140-mesh.

The particle size of a reinforcing phase is a critical design parameter that fundamentally dictates the quality of the fiber–matrix interface and composite performance. To elucidate the structure–property relationships in the PLA/DCS system, the interfacial morphology of the composites fabricated with 10 wt.% loading of four distinct DCS size fractions (80-, 100-, 120-, and 140-mesh) was investigated via scanning electron microscopy (Figure 3).

The analysis reveals that an optimal particle size window exists, with both extremes exhibiting deleterious microstructural features. The composite fabricated with the largest particles (80-mesh, Figure 3a) shows clear signs of poor compatibility. Many fibers are inadequately wetted by the PLA melt, resulting in weak interfacial adhesion evidenced by distinct microgaps and debonding at the fiber–matrix boundary. The fibers appear to be merely attached to the PLA surface rather than fully embedded. This phenomenon aligns with the theoretical framework by Gad et al. [32], who proposed that for large particles, processing shear forces are often insufficient to overcome the polymer’s surface tension and fully encapsulate the filler, transforming the intended reinforcement into stress-concentrating defects.

Conversely, the composite with the finest particles (140-mesh, Figure 3d) suffered from severe agglomeration. The high-surface-area particles clustered into large, irregular domains, creating a non-uniform surface. This behavior, as documented by Dave et al. [33], occurs when strong inter-particle van der Waals forces promote self-association that processing shear cannot overcome. These poorly penetrated agglomerates create internal voids that compromise structural integrity.

The intermediate particle sizes of 100-mesh (Figure 3b) and 120-mesh (Figure 3c) yielded marked improvements in interfacial quality. This observation aligns with established principles in composite science. Leu et al. [34] documented in their comprehensive review on wood–plastic composites that reducing the filler particle size, particularly below 125 μm, significantly enhances both tensile and flexural strength due to increased specific surface area, which promotes effective wetting and efficient stress transfer.

While the 100-mesh sample showed good integration, the 120-mesh DCS (125 μm) achieved visibly superior interfacial fusion (Figure 3c). The DCS particles are homogeneously embedded with seamless, indistinct interfaces between the filler and polymer, signifying a robust physical linkage essential for mechanical performance. However, the 120-mesh particle size represents an optimal balance: sufficiently fine to maximize the interfacial surface area for strong adhesion, yet large enough to mitigate the severe agglomeration tendencies observed with 140-mesh particles. Based on this compelling morphological evidence, the 120-mesh particle size was identified as optimal and selected for all subsequent investigations.

**Figure 4:**
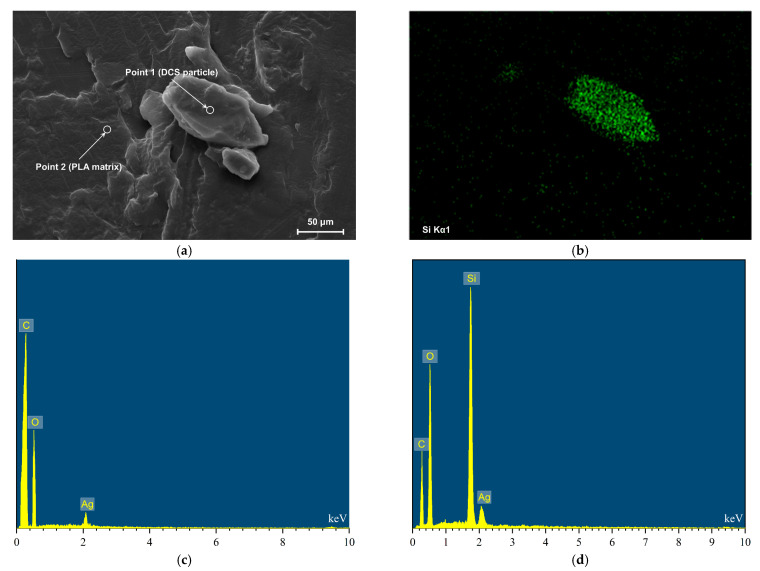
Elemental analysis confirming the two-phase structure of the PLA/DCS composite: (**a**) SEM micrograph of a representative interface selected for analysis; (**b**) corresponding elemental map for silicon (Si); (**c**) EDS spectrum of the PLA matrix; (**d**) EDS spectrum of the DCS particle.

To provide elemental validation for these morphological observations and confirm the successful incorporation of the distinct phases, Energy-Dispersive X-ray Spectroscopy (EDS) was performed. Two specific points were selected for analysis: Point 1 on a DCS particle and Point 2 on the PLA matrix (Figure 4a).

The EDS spectrum from Point 2 (PLA matrix, Figure 4c) revealed characteristic peaks only for carbon (C) and oxygen (O), the constituent elements of polylactic acid. By contrast, the spectrum from Point 1 (DCS particle, Figure 4d) displayed an additional prominent silicon (Si) peak along with C and O. The elemental mapping for silicon (Figure 4b) demonstrates that the Si signal is precisely confined to the geometric boundaries of the DCS particles, with no detectable signal in the surrounding PLA matrix.

The presence of silicon is intrinsic to gramineous plants like corn, which absorb silicic acid from soil and deposit it as amorphous hydrated silica (SiO_2_·nH_2_O) within their cellular structures, forming microscopic phytoliths [35]. These biogenic silica structures resist both enzymatic digestion and mild alkali treatment, remaining as a signature elemental tracer within the DCS. The clear elemental distinction between the Si-containing DCS particles (Point 1) and the Si-free PLA matrix (Point 2), combined with the visually seamless interface observed in the SEM, confirms successful and intimate incorporation of the DCS particles into the PLA matrix, forming a well-defined, two-phase composite material.

#### 3.1.3. Effect of DCS Content on Composite Microstructure

**Figure 5:**
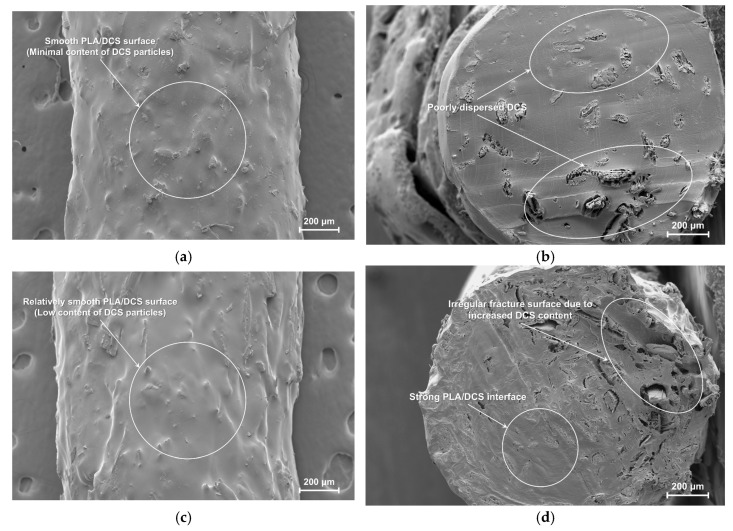

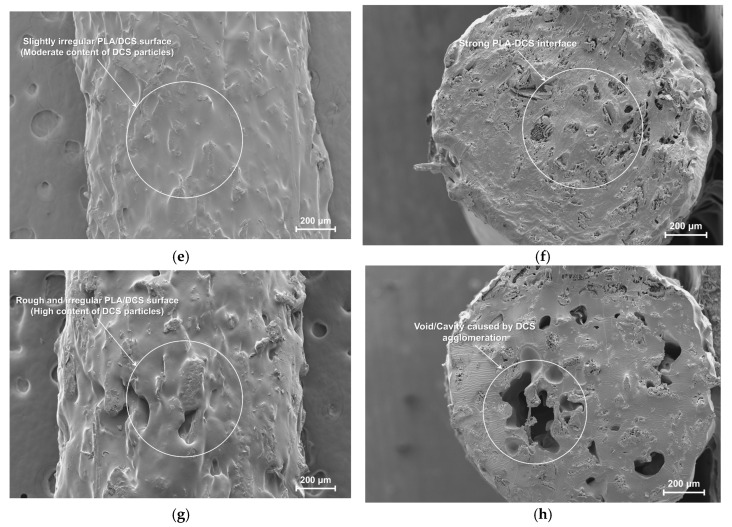
SEM micrographs showing the effect of filler content on the PLA/DCS composite microstructure: (**a**) surface and (**b**) cryo-fractured surface of 5 wt.% composite; (**c**) surface and (**d**) cryo-fractured surface of 10 wt.% composite; (**e**) surface and (**f**) cryo-fractured surface of 15 wt.% composite; (**g**) surface and (**h**) cryo-fractured surface of 20 wt.% composite.

Having established 120-mesh as the optimal particle size for achieving superior interfacial fusion, the subsequent critical step was to determine the ideal filler loading concentration. The weight fraction of the particulate reinforcement is a paramount parameter that profoundly influences the rheological properties during processing and the ultimate microstructural integrity of the final composite. To systematically investigate this, composites were fabricated with varying weight percentages of 120-mesh DCS particles: 5, 10, 15, and 20 wt.%. The external surfaces and cryo-fractured cross-sections of the resulting 3D-printed specimens were meticulously examined using SEM to evaluate particle dispersion, interfacial quality, and overall structural integrity (Figure 5).

At a low loading of 5 wt.% (Figure 5a,b), the DCS particles are sparsely and somewhat randomly distributed within the continuous PLA matrix. Due to the minimal filler content, the external surface of the composite (Figure 5a) remains relatively smooth. However, the cryo-fractured cross-section (Figure 5b) reveals a non-uniform morphology where the fracture plane is dominated by large, smooth regions characteristic of the brittle fracture of neat PLA, interspersed with areas containing isolated DCS particles. This indicates that the low population density of the particles prevents the formation of an effective, interconnected network for stress transfer. Consequently, the mechanical response at this loading is expected to be largely governed by the matrix itself, with the DCS particles acting more as passive inclusions than as a functional reinforcement.

A transition to a remarkably uniform and robust microstructure is observed at a 15 wt.% filler loading (Figure 5e,f). At this concentration, the system achieves an optimal equilibrium. The external surface of the printed part (Figure 5e) is notably smooth, indicating that the melt viscosity remained within a favorable range for achieving excellent flow and consolidation during Fused Deposition Modeling. More importantly, the cryo-fractured surface (Figure 5f) reveals a densely packed and well-integrated structure where the DCS particles are homogeneously dispersed and intimately encapsulated by the PLA matrix. The interface between the filler and matrix is seamless and indistinct, with no evidence of particle pull-out or interfacial debonding. This ideal microstructure is a prerequisite for efficient load transfer, a principle thoroughly established in the foundational work on wood–plastic composites by Schwarzkopf et al. [36].

In stark contrast, a further increase in filler content to 20 wt.% resulted in a marked deterioration of the composite microstructure. The high concentration of solid particles acts as a significant rheological impediment, substantially increasing the melt viscosity of the blend. This prevents uniform dispersion during extrusion, leading to the formation of large particle agglomerates, which are clearly visible on the irregular external surface (Figure 5g) and within the cryo-fractured cross-section (Figure 5h). This phenomenon, where filler particles transition from reinforcement to defect sites, stands in agreement with the theoretical models proposed by Ueno et al. [37], which predict the onset of agglomeration when inter-particle forces overcome the dispersive shear forces during melt compounding. These clusters are poorly wetted by the viscous polymer, creating significant interfacial voids and weak points within the structure. This finding, where processing physics negate the benefits of chemical surface treatments, aligns with the work of Atta et al. [38], who proposed that, at very high filler loadings, the dominant failure mechanism shifts from interfacial debonding to processing-induced agglomeration.

Based on the morphological evidence indicating superior dispersion and interfacial fusion, the 15 wt.% loading was identified as the most promising formulation. To rigorously validate this observation and fully characterize the structure-property relationships across the entire compositional range, all formulations were subsequently subjected to the full suite of physical and mechanical tests.

### 3.2. Density and Porosity Analysis

**Figure 6:**
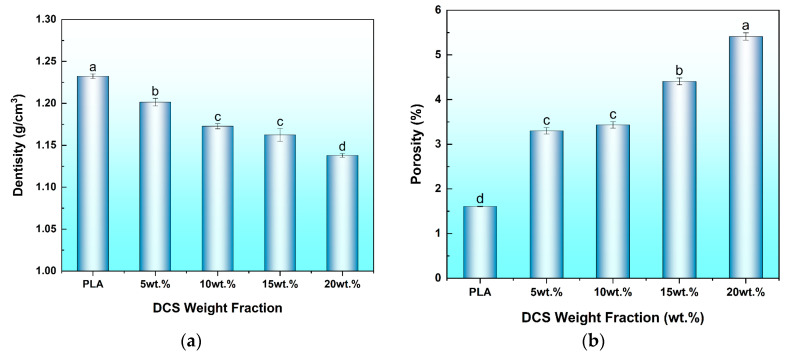
Physical properties of 3D-printed PLA/DCS composites as a function of fiber content: (**a**) experimental density; (**b**) calculated porosity. Different letters (e.g., a, b, c) indicate significant differences among the groups (*p* < 0.05).

The bulk density and internal porosity of additively manufactured components are paramount physical properties, as they directly govern the final part weight and, more critically, the structural integrity by controlling the prevalence of performance-limiting defects. An analysis of the experimental density and calculated porosity was therefore conducted to quantify the effects of DCS fiber incorporation on the quality of the 3D-printed composites, with the results presented in Figure 6.

The data reveal a systematic and advantageous reduction in the experimental density of the composites as a function of increasing DCS fiber content. The neat PLA specimen exhibited a density of 1.232 g/cm^3^, a value consistent with its specified grade. Upon reinforcement, the density decreased in a near-linear fashion, reaching a minimum of 1.138 g/cm^3^ for the 20 wt.% composite. This trend is a direct consequence of the rule of mixtures, whereby the denser PLA matrix (*ρ* ≈ 1.24 g/cm^3^) is progressively replaced by the significantly lighter lignocellulosic DCS fibers. This outcome is highly desirable for the development of lightweight materials, a primary objective in many advanced engineering applications, and aligns with the lightweighting goals pursued in numerous studies on natural fiber composites, as researched by de Melo et al. [39]. It is noteworthy that this advantageous reduction in density is often accompanied by an increase in porosity, a dual-trend also systematically documented by Awad et al. [40] in their study on date palm fiber/PLA composites.

Conversely, the analysis of porosity, a measure of the void content within the printed specimens, provides critical insight into the processability of the composite formulations and the quality of the resulting microstructure. The neat PLA specimen displayed a baseline porosity of 1.61%, a value that is not unexpected. This baseline porosity is a well-documented characteristic of the FDM process itself, originating from microscopic imperfections such as incomplete fusion between adjacent rasters and layers, a phenomenon thoroughly investigated by Tronvoll et al. [41].

The introduction of DCS fibers, however, resulted in a monotonic increase in porosity, a trend attributable to a combination of distinct physical mechanisms. A primary contributor is the formation of microscopic voids at the fiber–matrix interface. While the surface treatments successfully enhanced mechanical interlocking, the absence of perfect chemical continuity can lead to the entrapment of air during the high-shear melt extrusion and printing processes. Furthermore, the inherent cellular structure of the lignocellulosic fibers, including their natural lumens, introduces a degree of intrinsic porosity to the system.

Most significantly, the porosity trend strongly corroborates the microstructural evidence of processing challenges at high filler loadings. The porosity increased moderately to 4.41% at a 15 wt.% loading, but then exhibited a pronounced jump to 5.41% at 20 wt.%. This sharp increase is a clear indicator of rheological impediment. As fiber concentration rises, the melt viscosity of the composite increases substantially, which hinders uniform flow and prevents the polymer from effectively wetting the fibers and consolidating into a dense structure. This phenomenon, where rheological limitations at high filler loadings lead to a sharp increase in void content, has been theoretically modeled and experimentally confirmed by Mukhtar et al. [42] in other highly filled polymer systems. The fiber agglomeration observed Via SEM at 20 wt.% loading is a direct manifestation of this poor consolidation, creating larger inter-particle voids that the viscous melt cannot penetrate. As these voids act as stress concentration sites, their proliferation at 20 wt.% loading is a definitive sign of compromised mechanical integrity, further validating that the 15 wt.% formulation represents the optimal threshold for achieving effective reinforcement without inducing catastrophic processing defects.

### 3.3. Water Absorption Behavior

**Figure 7:**
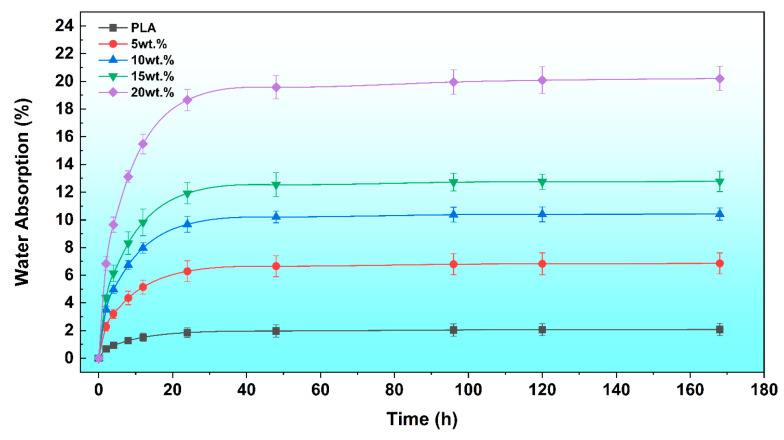
Water absorption curves for neat PLA and PLA/DCS composites with varying fiber content (0, 5, 10, 15, and 20 wt.%) over a 168 h immersion period.

The long-term durability and dimensional stability of the composites were evaluated by quantifying their water absorption kinetics. The water absorption kinetics of the neat PLA and PLA/DCS composites over a 168 h immersion period are presented in Figure 7. The results confirm that the incorporation of ruminant-digested corn stover (DCS) fundamentally alters the hydrophobic characteristic of the polylactic acid (PLA) matrix. The neat PLA specimen, serving as a baseline, demonstrated excellent water resistance, with its equilibrium water uptake plateauing at a minimal 2.08%.

The introduction of DCS fibers, which are rich in hydrophilic hydroxyl groups, creates pathways for moisture ingress. This behavior is a well-documented characteristic of natural fiber composites, as extensively reviewed by Chandrasekar et al. [43]. Consequently, the equilibrium water absorption increased with fiber fraction. However, the nature of this increase provides critical insight into the composite’s internal structure. The incremental rise in water absorption between the 5 wt.% (6.85%) and 10 wt.% (10.42%) composites was 3.57%, which then decreased to just 2.36% for the increment between the 10 and 15 wt.% (12.78%) composites. This diminishing marginal increase suggests that at loadings up to 15 wt.%, the PLA matrix achieves relatively effective encapsulation of the well-dispersed fibers, moderating their access to environmental moisture.

In stark contrast, a profound shift in behavior occurs at the 20 wt.% loading. The equilibrium water absorption surged to 20.21%, representing a massive 7.43% increase from the 15 wt.% sample. This disproportionate leap is not attributable to the simple addition of more fiber; rather, it signals a fundamental change in the water transport mechanism. This finding powerfully corroborates the morphological and porosity analyses (Section 3.1.3 and Section 3.2), which identified significant fiber agglomeration and a sharp rise in void content at this concentration. This defective microstructure creates an interconnected network of interfacial gaps and voids, which facilitates rapid water ingress Via capillary action. Therefore, the water absorption data provides compelling, independent validation that exceeding a 15 wt.% loading threshold critically compromises the composite’s structural integrity, reinforcing its selection as the optimal formulation.

### 3.4. Tensile Properties

**Figure 8:**
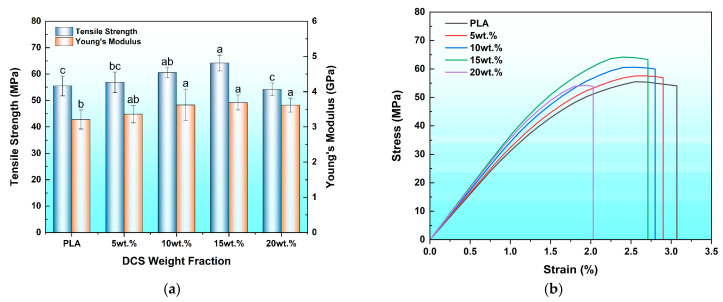
Tensile properties of 3D-printed PLA/DCS composites as a function of fiber content: (**a**) tensile strength and Young’s modulus; (**b**) Representative stress–strain curves. Different letters indicate significant differences among the groups (*p* < 0.05).

The quasi-static tensile response of a composite provides fundamental insights into its load-bearing capacity and failure mechanisms, serving as a primary indicator of reinforcement efficacy. The tensile properties of the 3D-printed PLA/DCS composites were profoundly influenced by the particulate weight fraction, revealing an optimal loading concentration where mechanical performance is maximized before being compromised by processing-induced defects (Figure 8).

The tensile strength of the composites exhibited a distinct non-monotonic dependence on filler concentration, a trend that underscores the critical interplay between interfacial quality and particle dispersion (Figure 8a). The strength increased from 55.51 MPa for the neat PLA matrix to a peak value of 64.17 MPa at a 15 wt.% loading, representing a notable 15.6% enhancement. This improvement is not attributed to the intrinsic strength of the DCS particles, but rather to the excellent interfacial adhesion achieved at this concentration. This mechanism is well-understood in the field of reinforced bioplastics; as the comprehensive research by Mohit et al. [44] shows, the quality of the interfacial bond is a primary determinant of a composite’s ability to effectively transfer stress and resist failure. Notably, this principle of reinforcement extends beyond monolithic composites. For instance, Vinod et al. [45] recently demonstrated a similar positive reinforcement trend in 3D-printed hybrid natural fiber sandwich composites, reporting significant improvements in their thermomechanical properties. Their work, while focusing on a different structural design, further validates the potential of natural fillers to effectively enhance the PLA matrix. This principle, confirmed by the seamless interface observed in the SEM analysis (Section 3.1.3), is what allows the composite to delay the onset of catastrophic failure.

The incorporation of DCS fibers resulted in a significant improvement in the material’s stiffness, as evidenced by the Young’s modulus data (Figure 8a). The modulus systematically increased with fiber content, rising from 3.21 GPa for neat PLA to a peak value of 3.69 GPa at a 15 wt.% loading, representing a 15% enhancement. This trend, which is consistent with the flexural modulus results, confirms the effective reinforcing role of the rigid lignocellulosic fibers within the PLA matrix. Interestingly, a further increase in fiber content to 20 wt.% led to a slight decrease in the modulus to 3.62 GPa. While the modulus at 20 wt.% remains significantly higher than that of neat PLA, this slight downturn indicates that a critical threshold has been reached. At this high loading, the negative effect of increased porosity and particle agglomeration, as observed in Section 3.1.3 and Section 3.2, begins to counteract the stiffening effect of the added fibers. This behavior contrasts with the sharp drop observed in tensile strength at the same concentration, highlighting that the material’s stiffness is less sensitive to processing-induced defects than its ultimate strength.

However, a further increase in filler content to 20 wt.% precipitated a sharp strength reduction to 54.20 MPa, a value below that of the unreinforced matrix. This reversal demonstrates that a critical threshold was surpassed, where the benefits of good adhesion are completely negated by processing-induced defects. As confirmed by the morphological and porosity analyses, the significant particle agglomeration and void formation prevalent at this high loading create numerous internal flaws. These agglomerates, which are poorly wetted by the viscous polymer, function as potent stress concentrators that initiate premature failure. This direct link between poor dispersion and the creation of performance-limiting stress concentrations is a critical concept, recently reinforced by Sansone et al. [46], who definitively showed that such agglomeration phenomena in hierarchically structured composites lead to localized high-stress regions that govern material failure.

The transition in the material’s failure mode from ductile to more brittle behavior is clearly illustrated by the representative stress–strain curves presented in Figure 8b. The neat PLA curve exhibits the most pronounced plastic deformation region before fracture, corresponding to the highest elongation at break (3.07%). With the incorporation of rigid DCS fibers, the material becomes progressively more brittle. This is visually confirmed by the decreasing strain at fracture for the 5 wt.%, 10 wt.%, and 15 wt.% composites. This classic embrittlement occurs because the rigid filler phase constrains the segmental mobility of the polymer chains, restricting their ability to deform plastically under load. The curve for the 20 wt.% composite shows a further, abrupt loss of ductility, with elongation plummeting to just 2.03%. This is not merely an extension of the stiffening trend but a direct consequence of the compromised microstructural integrity at this high loading. The pervasive agglomeration and increased porosity, as discussed previously, act as pre-existing flaws that initiate cracks, leading to premature, brittle failure with minimal plastic deformation. This shift from a more ductile, matrix-dominated failure to a defect-initiated brittle fracture is a key finding of the tensile analysis.

Collectively, the tensile evaluation provides compelling mechanical evidence that corroborates the microstructural analysis. The data unequivocally identifies the 15 wt.% formulation as the optimal system, as it successfully leverages strong interfacial adhesion to enhance strength without introducing the catastrophic processing defects that dominate at higher concentrations. This specific loading maximizes tensile strength while avoiding the abrupt loss of ductility and structural integrity observed at the 20 wt.% threshold, where particle agglomeration and increased porosity become the dominant failure mechanisms.

### 3.5. Flexural Properties

**Figure 9:**
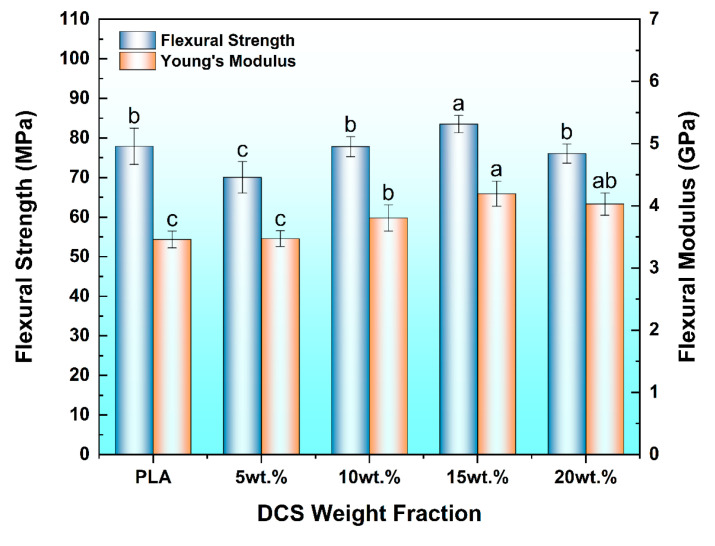
Flexural properties of 3D-printed PLA/DCS composites as a function of fiber content. Different letters indicate significant differences among the groups (*p* < 0.05).

The flexural properties of the composites, which are paramount for structural applications, revealed a complex interplay between reinforcement and processing-induced microstructural features (Figure 9). The flexural modulus, representing the material’s stiffness, demonstrated the most direct reinforcing effect of the rigid DCS fibers.

As anticipated, the modulus increased systematically from 3.46 GPa for neat PLA to a maximum of 4.19 GPa at a 15 wt.% loading—a substantial 21.1% enhancement. This classic stiffening behavior, which aligns with the theoretical framework established by Prabhakar et al. [47] for particulate-filled polymers, confirms that the DCS fibers effectively constrain the polymer matrix and resist bending deformation. The slight downturn in modulus at 20 wt.% is a clear indicator that the increased porosity detailed in Section 3.2 has begun to compromise the composite’s effective load-bearing cross-section.

In stark contrast, the flexural strength—the material’s ultimate resistance to failure in bending—followed a more telling non-monotonic path. Critically, the initial addition of 5 wt.% DCS resulted in a decrease in flexural strength relative to the neat PLA matrix. This initial detrimental effect is a well-understood phenomenon; at low concentrations, the sparsely distributed particles fail to form an effective stress-transfer network and instead act primarily as stress-concentrating discontinuities within the matrix, a concept previously modeled by Ali et al. [48].

The pivotal transition in mechanical behavior occurred as the loading increased. The flexural strength only recovered and ultimately surpassed that of the unreinforced PLA matrix at the 15 wt.% concentration, where it reached a peak value of 83.50 MPa. This finding is of central importance, as it pinpoints the precise composition where the system achieves microstructural and mechanical optimality. The principle that adding plant fibers can enhance the flexural properties of FDM-printed PLA is supported by existing literature. For instance, in a study on sugarcane bagasse fiber composites, Liu et al. [49] demonstrated a clear reinforcing effect on material stiffness, reporting that the flexural modulus of a 15 wt.% composite reached approximately 2.7 GPa—a significant 28.6% increase over the pure PLA matrix. As confirmed by our SEM analysis (Section 3.1.3), it is at this specific loading that the system achieves an ideal microstructure, characterized by uniform particle dispersion and maximized interfacial adhesion. This optimized microstructure is critical as it minimizes particle-induced stress concentration effects while facilitating highly efficient load transfer from the matrix to the fibers, thereby synergistically enhancing the composite’s strength. This principle—that the ultimate strength of a composite fabricated Via fused filament fabrication is fundamentally governed by its interfacial integrity—has been powerfully reinforced in recent studies. For instance, the work of Khan et al. [50] on complex tri-material systems demonstrated that achieving a robust interface is the paramount factor in realizing the mechanical potential of the composite structure.

The subsequent sharp decline in strength at 20 wt.% provides definitive evidence that this optimal threshold has been exceeded. As established in our morphological and porosity analyses, the high melt viscosity at this loading leads to significant fiber agglomeration and void formation. These defects act as potent stress risers, negating the benefits of reinforcement and causing premature failure under flexural load. This behavior underscores that at 15 wt.% loading, the composite is a uniformly reinforced material, while at 20 wt.%, it has transitioned into a flawed structure dominated by processing-induced defects.

### 3.6. Impact Strength

**Figure 10:**
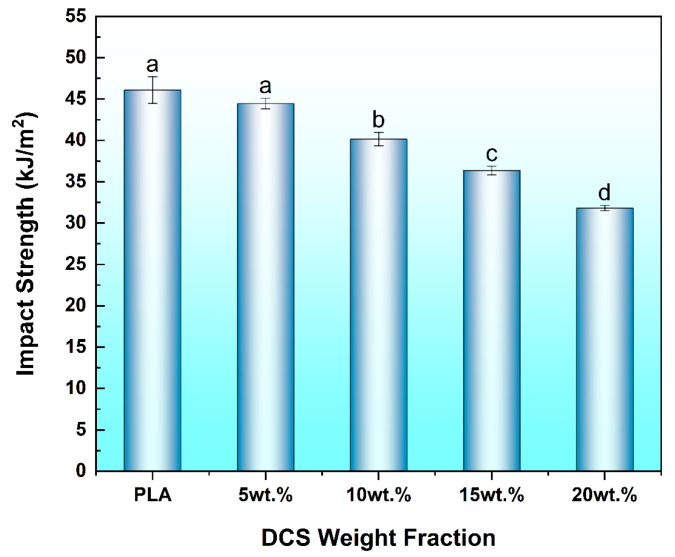
Notched Izod impact strengths of 3D-printed PLA/DCS composites as a function of fiber content. Different letters indicate significant differences among the groups (*p* < 0.05).

The capacity of the composite to absorb energy under sudden, high-velocity loading was quantified Via Notched Izod impact testing, a high-strain-rate test that measures a material’s resistance to brittle fracture. The results, presented in Figure 10, reveal a systematic and statistically significant degradation of toughness as a function of fiber content. The neat PLA matrix exhibited an impact strength of 46.07 kJ/m^2^, which monotonically decreased to a minimum of 31.81 kJ/m^2^ at the highest filler loading of 20 wt.%. This trend indicates that the composite system is fundamentally ill-suited for applications involving impact, a behavior rooted in the dynamic response of both the polymer matrix and the filler phase.

The primary cause for this embrittlement is the inherent strain-rate sensitivity of the PLA matrix. Under the rapid loading conditions of an impact event, there is insufficient time for the polymer chains to undergo the segmental relaxation and reptation that enable ductile energy dissipation, causing the matrix to respond in a glassy, brittle fashion. This embrittlement is a well-documented response in natural-fiber-reinforced polymers under high-strain-rate loading, as reviewed by Khieng et al. [51], where the rigid filler phase restricts the segmental mobility of the surrounding polymer chains. This trend is consistent with other studies, such as Gozdecki et al. [52], who found that increasing tall wheatgrass filler in PLA to 50% caused a 71% reduction in impact strength due to void formation and poor adhesion. The DCS fibers further exacerbate this intrinsic brittleness by acting as stress concentrators, creating localized high-stress regions that initiate micro-cracks with minimal energy input.

This transition from quasi-static strength to dynamic brittleness is a defining characteristic of many engineering materials, and the failure mechanism observed here aligns with the frameworks for dynamic cracking in brittle systems. As Forquin [53] has extensively reviewed, brittle materials exhibit a strong strain-rate sensitivity, where failure under impact is governed by the rapid activation and propagation of cracks from a population of inherent flaws. In the PLA/DCS system, the DCS particles themselves, along with the interfacial voids and agglomerates that become prevalent at higher loadings (as detailed in Section 3.1.3 and Section 3.2), serve as this population of critical flaws. The fiber–matrix interface, rather than providing energy-dissipating mechanisms like fiber pull-out, becomes a preferential, low-energy pathway for crack propagation. This leads to premature, catastrophic failure and explains the observed monotonic decrease in absorbed energy. Ultimately, the data highlight a classic engineering trade-off: the significant enhancements in quasi-static strength and stiffness are achieved at the direct expense of the material’s toughness.

## 4. Conclusions

This study systematically demonstrated the feasibility of utilizing ruminant-digested corn stover (DCS), an underutilized agricultural waste, as a novel reinforcing filler for polylactic acid (PLA) to create a sustainable, 3D-printable biocomposite. Through a comprehensive evaluation of material preparation, processing parameters, and the resulting composite properties, this research successfully identified an optimal formulation and established a viable pathway for the valorization of DCS within a circular bioeconomy framework. The key findings and their implications are summarized as follows:

Morphological analysis definitively established that a fiber particle size of 120-mesh is optimal for integration into the PLA matrix. This size achieved a superior, seamless fusion with the polymer, ensuring a well-integrated composite structure essential for effective reinforcement, whereas larger particles showed poor adhesion and smaller ones tended to agglomerate.

A fiber loading of 15 wt.% was identified as the optimal concentration, achieving peak mechanical performance. At this loading, the composite exhibited a 15.6% increase in tensile strength (to 64.17 MPa) and a substantial 21.1% increase in flexural modulus (to 4.19 GPa) compared to neat PLA, confirming effective stress transfer from the matrix to the well-dispersed fibers.

Consistent with the behavior of many particle-reinforced composites, the enhanced stiffness and strength were accompanied by a reduction in impact toughness. The incorporation of rigid DCS fibers restricted the mobility of PLA polymer chains, a classic mechanism that increases brittleness while improving the strength and modulus.

A significant advantage of incorporating DCS was the creation of a lightweight composite, with density decreasing as fiber content increased. Concurrently, the hydrophilic nature of the lignocellulosic fibers led to increased water absorption, a characteristic trade-off for natural fiber composites. The disproportionately high absorption at 20 wt.% was directly linked to structural defects, further validating 15 wt.% as the superior formulation.

In conclusion, this research represents a significant contribution to the field of sustainable materials by successfully transforming a problematic agricultural residue into a value-added functional filler for advanced manufacturing. The development of a 3D-printable PLA/DCS biocomposite with significantly enhanced tensile strength and flexural modulus not only demonstrates a novel industrial application for this abundant biomass but also contributes directly to the principles of a circular bioeconomy. However, it is crucial to acknowledge that the addition of DCS fibers introduces potential disadvantages rooted in the known sensitivities of PLA-based materials. The incorporation of hydrophilic lignocellulosic fibers leads to a predictable increase in water absorption, which can accelerate the material’s aging and embrittlement over time—a common challenge for PLA filaments. This is compounded by the significant reduction in impact strength, a critical trade-off for applications requiring high toughness. These challenges, however, define clear directions for future work. Subsequent research should focus on advanced interfacial engineering and the incorporation of stabilizing additives to mitigate moisture-induced degradation, thereby enhancing long-term durability and impact toughness. This study lays a robust foundation, opening new possibilities for the design and fabrication of next-generation, eco-friendly composites that are both high-performance and fully integrated into a sustainable resource cycle.

## Figures and Tables

**Table 1:** Parameters for statistical analysis.

Parameter	Specification
Data presentation	Mean ± standard deviation (SD)
Replicates	Minimum of five (n ≥ 5) per formulation
Software	SPSS 26.0 statistics
Primary test	One-way analysis of variance (ANOVA)
Post hoc test	Fisher’s least significant difference (LSD)
Significance level	*p* < 0.05

## Data Availability

The original contributions presented in this study are included in the article. Further inquiries can be directed at the corresponding authors.
